# Hemodynamic management, including for left ventricular outflow tract obstruction, in an infant with ACTH-independent Cushing syndrome undergoing adrenal tumor removal: a case report

**DOI:** 10.1186/s40981-026-00868-2

**Published:** 2026-07-11

**Authors:** Mariko Saito, Maiko Hosokawa, Kenichi Masui

**Affiliations:** 1 Department of Anesthesiology, Showa Medical University School of Medicine, Tokyo, Japan; 2 Department of Anesthesiology, Japanese Red Cross Medical Center, Tokyo, Japan; 3 Department of Anesthesiology, Yokohama City University School of Medicine, 3-9 Fukuura, Kanazawa-ku, Yokohama, 236-0004 Japan

**Keywords:** Adrenocorticotropic hormone -independent Cushing syndrome, Left ventricular outflow tract obstruction, Infant, Adrenal tumor, Adrenalectomy

## Abstract

**Background:**

Adrenocorticotropic hormone (ACTH)-independent Cushing syndrome is extremely rare in children, with moon face, central obesity, buffalo hump, and hypertension as typical characteristics. We focused on perioperative hemodynamic management because this patient had left ventricular outflow tract obstruction (LVOTO), which is a rare sequela.

**Case description:**

A 6-month-old boy with ACTH-independent Cushing syndrome and sustained hypertension, with a cortisol level of 49.0 µg·dL^− 1^, was scheduled to undergo open adrenal tumor resection. Since the preoperative echocardiogram showed left ventricular hypertrophy and LVOTO, preoperative fluid loading was performed over 2 days to optimize fluid volume. For anesthesia induction, the ramp position helped maintain airway patency. Intraoperative and postoperative hemodynamics were generally stable, and hypertension during tumor manipulation was managed by titrating sevoflurane concentration.

**Conclusions:**

An excessive cortisol level with Cushing syndrome can rarely cause LVOTO in an infant. Not only airway management but also hemodynamic management, including preoperative management, would be essential.

**Supplementary Information:**

The online version contains supplementary material available at https://doi.org/10.1186/s40981-026-00868-2.

## Background

Adrenocorticotropic hormone (ACTH)-independent Cushing syndrome in children under 15 years has an incidence of 0.2–0.5 per million per year [1–3]. Treatment typically involves adrenalectomy. Anesthetic management for the surgery is often complicated by several challenges related to hemodynamics, perioperative endocrine functions, and airway management [4].

In Cushing syndrome patients, hypertension is a common complication. Additionally, steroid-induced myocardial hypertrophy is known in preterm infants. Previous articles reported that myocardial hypertrophy was diagnosed in 40% to 57% ventilated infants who received glucocorticoids [5]. However, myocardial hypertrophy is rare in a patient with an adrenal tumor [6]. When the continuous excessive cortisol release by an adrenal tumor causes myocardial hypertrophy and left ventricular outflow tract obstruction (LVOTO), perioperative fluid management would be required [7] to avoid hypovolemia. LVOTO reduces left ventricular end-diastolic volume, increases contractility and outflow tract flow velocity, thereby worsening the pressure gradient [8, 9]. In infants, who have limited circulating blood volume, even modest intraoperative blood loss can result in critical hypovolemia and precipitate dynamic LVOTO. Additionally, general anesthetics, such as sevoflurane, decrease systemic vascular resistance [10]. Therefore, careful perioperative volume management is essential to maintain hemodynamic stability during surgery [11]. 

We focused on perioperative hemodynamic management for an infant undergoing an open adrenalectomy with ACTH-independent Cushing syndrome with sequelae of hypertension and LVOTO. This case report was prepared in accordance with the CARE guidelines [12]. 

## Case description

**Fig. 1:**
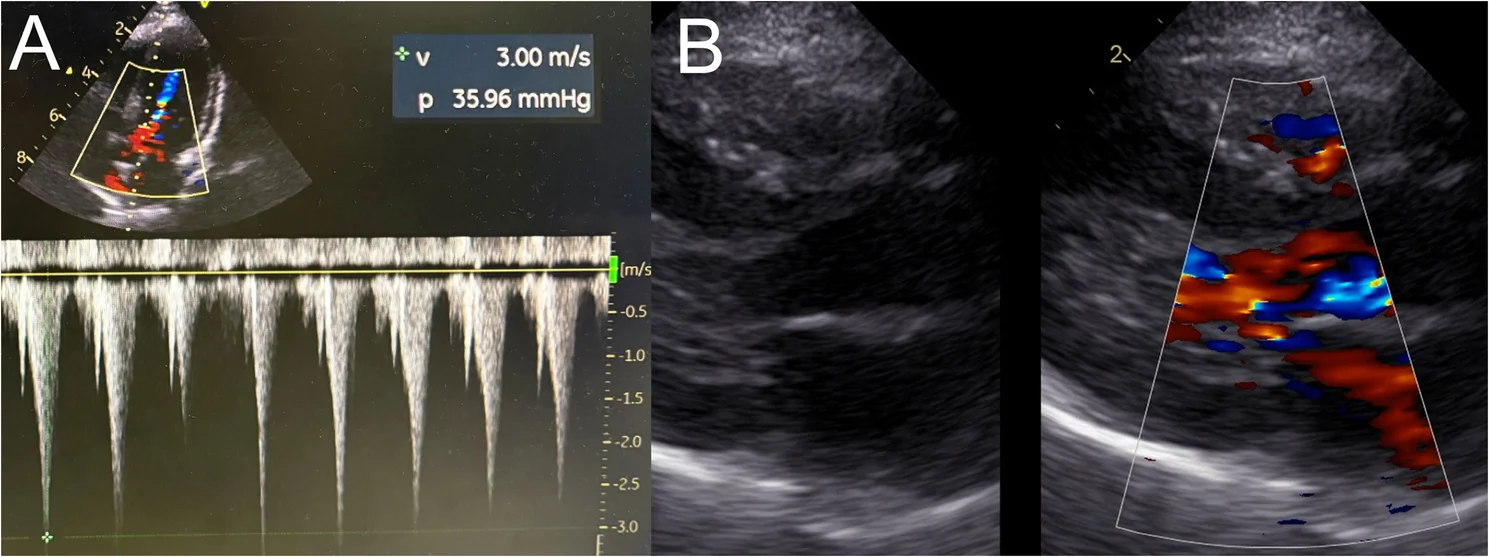
Transthoracic echocardiography images before operation. These images were obtained during transthoracic echocardiography performed in the echocardiography laboratory six days before surgery. **a** Apical view with continuous-wave Doppler across the left ventricular outflow tract, demonstrating a peak velocity of 3.0 m/s. **b** Parasternal long-axis view showing marked left ventricular hypertrophy

A 6-month-old boy (58.6 cm, 7.4 kg) with a short stature (**−** 2.0 SD) and moon face was diagnosed with ACTH-independent Cushing syndrome and a 6 cm right adrenal tumor. He presented with hypertension (systolic blood pressure 140–160 mmHg) and elevated preoperative serum cortisol levels (49.0 µg·dL^− 1^). Echocardiography showed hyperkinetic left ventricular motion, concentric hypertrophy, and left ventricular outflow tract obstruction (LVOTO) with a peak velocity of 3.0 m·s^− 1^ (Fig. 1). The preoperative highest peak velocity was 3.3 m·s^− 1^. A markedly elevated plasma norepinephrine level (1.12 ng·mL^− 1^) was observed. As it was difficult to control hypertension with internal medicines such as 33 mg·kg^− 1^·day^− 1^ metyrapone and 0.15 mg·kg^− 1^·day^− 1^ nifedipine, he was scheduled for surgical resection of the adrenal tumor.

Preoperative fluid therapy was implemented to prevent severe hypotension during surgery caused by LVOTO and dehydration. The administered fluid volumes were 70 ml·kg^− 1^·day^− 1^ two days before, 100 ml·kg^− 1^·day^− 1^ the day before, and 130 ml·kg^− 1^·day^− 1^ on the day of surgery. For perioperative steroid coverage, intravenous administration of hydrocortisone at 100 mg·m^− 2^·day^− 1^ from three days before surgery until two days after surgery. For surgical anesthesia, we prepared phenylephrine, adrenaline, noradrenaline, atropine, and red blood cells, and planned arterial and central venous pressure monitoring.

**Fig. 2:**
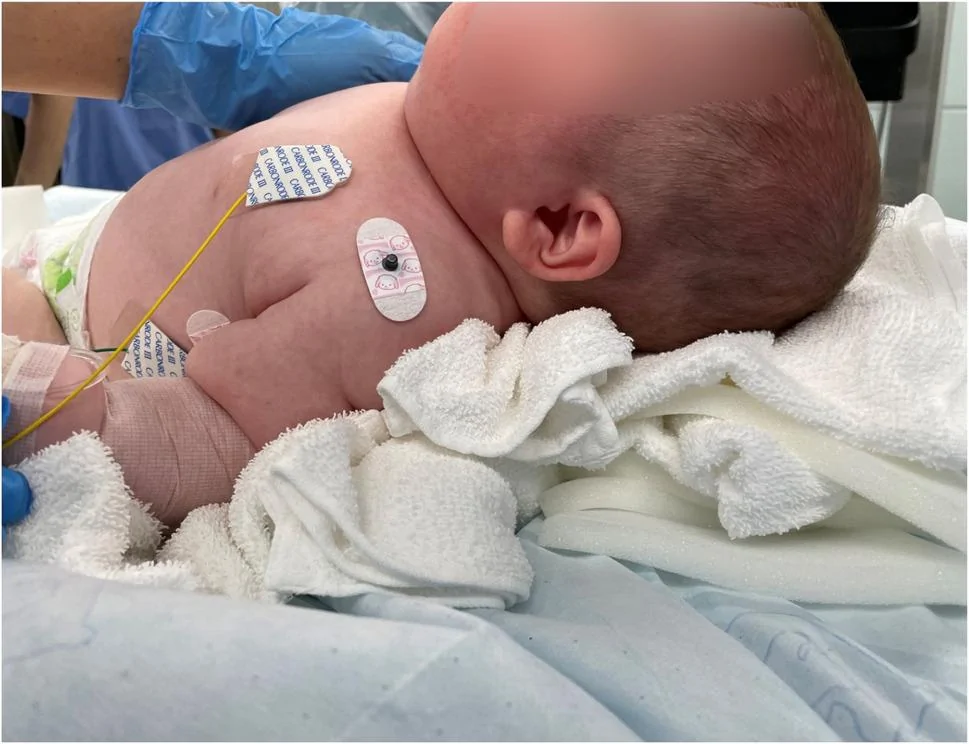
Patient appearance. Ramped position to maintain airway patency before anesthetic induction

**Fig. 3:**
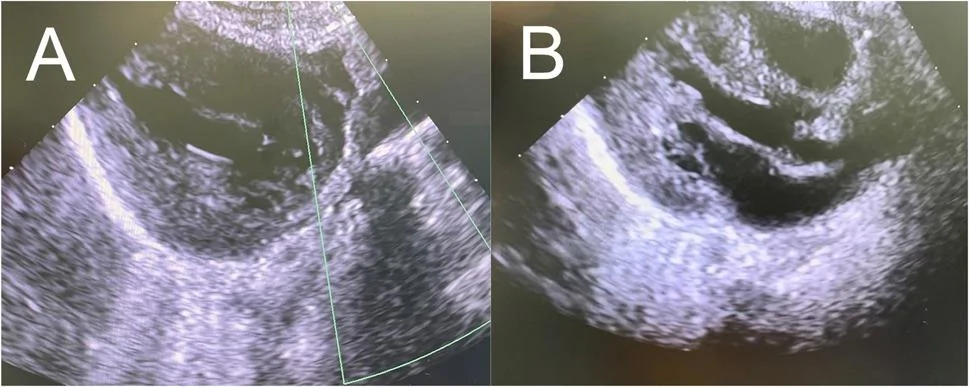
Transthoracic echocardiography after induction of anesthesia. The images were obtained after anesthetic induction and before surgical incision. **A** Parasternal short-axis view, **B** Parasternal long-axis view

After entering the operating room, he was placed in a ramp position (Fig. 2). General anesthesia was induced using intravenous midazolam 1 mg and rocuronium 10 mg while providing 100% oxygen with 2% sevoflurane via face mask. Two-person ventilation was appropriate because of his moon face. A 3.0 mm Microcuff^®^ endotracheal tube was smoothly inserted into the trachea using a size 1 Macintosh blade. The patient was carefully moved from the ramped position to supine, and we confirmed that the endotracheal tube position was appropriate. Then, radial arterial and internal jugular venous lines were inserted. Following preoperative fluid loading, transthoracic echocardiography (TTE) demonstrated no worsening of left ventricular outflow tract obstruction compared with the preoperative findings (Fig. 3).

**Fig. 4:**
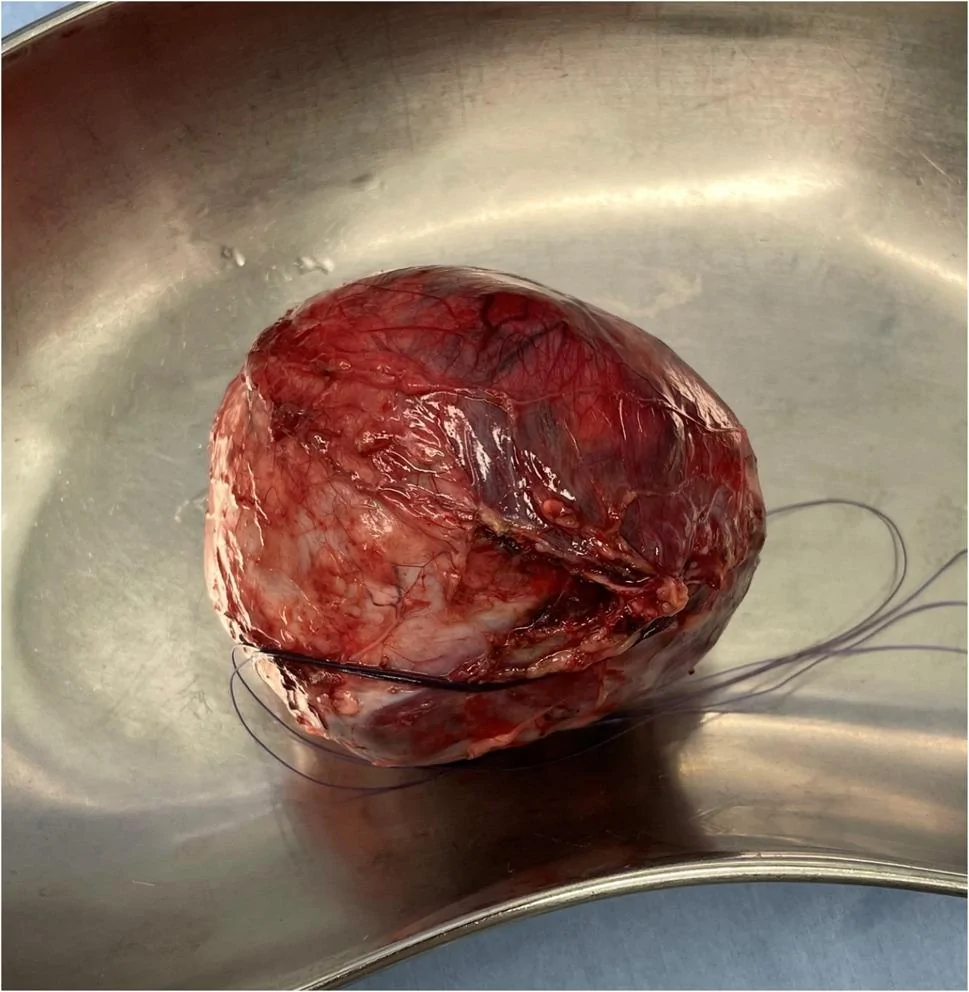
Resected adrenal tumor. The specimen measured 70 × 80 × 55 mm and weighed 125 g

Anesthesia was maintained with sevoflurane (1.3–2.0%) and remifentanil (0.08–0.3 µg·kg^− 1^·min^− 1^). Hydrocortisone infusion was initiated at 1.25 mg/h for steroid coverage. To maintain preload, 90 ml of 5% albumin was administered throughout general anesthesia. Systolic blood pressure (SBP) was maintained at approximately 130–140 mmHg before the adrenal tumor manipulation. During tumor removal, the SBP spiked to 220 mmHg with tachycardia. We administered sevoflurane at 5%, which reduced the SBP to approximately 120 mmHg within 5 min. After SBP was recovered, the sevoflurane concentration was reduced to 2%. Hemodynamics then remained stable. The resected adrenal tumor is shown in Fig. 4. Surgery was completed without complications, except for hypertension. TTE showed no change in LVOTO just after the surgery. Before confirming stable hemodynamics after surgery, the patient remained intubated with the administration of dexmedetomidine (0.5 µg·kg^− 1^·h^− 1^) and fentanyl (4 µg·kg^− 1^·h^− 1^). The patient was transferred to the ICU without being extubated. The anesthesia time was 4 h 7 min.

To treat SBP at approximately 130 mmHg after surgery, intravenous nicardipine was administered continuously until POD 2 in the ICU. The patient was sedated with midazolam at 0.12 mg·kg^− 1^·h^− 1^ and fentanyl at 4 µg·kg^− 1^·h^− 1^ until being extubated on POD 1. He received intravenous hydrocortisone at 100 mg·m^− 2^·day^− 1^ until POD 2, after which it was transitioned to oral administration upon discharge from the ICU on POD 3 with a decrease to 50 mg·m^− 2^·day^− 1^ between POD 3 and 5, 25 mg·m^− 2^·day^− 1^ during POD 6 and 8, and 15 mg·kg^− 1^·day^− 1^ on POD 9. The patient was discharged home on POD 14.

## Discussion

In this case, two challenges for hemodynamics were anticipated in managing (1) hemodynamic instability associated with left ventricular hypertrophy and potential worsening of the LVOTO; and (2) fluctuations in cortisol and catecholamine levels due to adrenal tumor manipulation. Elevated cortisol levels are known to induce myocardial hypertrophy and rarely to develop LVOTO [13]. Although metyrapone was administered for 10 days, the serum cortisol level did not recover to normal on the day of surgery due to persistent severe hypertension. If feasible, it would be preferable to normalize cortisol levels before surgery. In this case, serum cortisol levels normalized by POD 2, and transthoracic echocardiography demonstrated regression of myocardial hypertrophy. Follow-up echocardiography revealed that mild left ventricular wall thickening, but not LVOTO, persisted at one month.

This case exhibited LVOTO, which could be worsened by hypovolemia [14], but preoperative fluid therapy and intraoperative albumin administration [15] resulted in stable hemodynamics. Transesophageal echocardiography (TEE) may assist in hemodynamic evaluation, but it was not performed to avoid airway and esophageal injury [16]. Alternatively, perioperative point-of-care ultrasound was useful in this case.

Marked hypertension > 200 mmHg and tachycardia > 120 beats·min^− 1^ occurred during the adrenal manipulation, likely due to catecholamine release. To minimize the risk of severe hypotension due to tumor removal, sevoflurane and an ultra-short-acting β-blocker were considered. We selected sevoflurane for its rapid onset and offset, rather than a β-blocker, which may impair the compensatory hemodynamic response. Previously, esmolol was reported to be administered to counteract a hypertensive reaction during adrenal tumor manipulation in a three-year-old child with ACTH-independent Cushing syndrome [1]. Our preoperative fluid therapy and perioperative hydrocortisone infusion may have helped support hemodynamic stability, as inotropic agents were not required after the tumor resection [17]. A recent case report described significant hypotension after tumor resection, which was treated with an inotropic agent [1]. Preoperative and intraoperative fluid management and preparation of inotropic agents should be considered for children undergoing adrenal tumor resection for Cushing syndrome to address potential hemodynamic compromise after excision.

To manage hemodynamic factors, including cortisol-induced myocardial hypertrophy and rarely occurring LVOTO, in infants with Cushing syndrome, preoperative transthoracic echocardiography is essential. Preoperative management should include cortisol-lowering therapy with metyrapone, antihypertensive treatment such as calcium channel blockers, and adequate volume loading to optimize perioperative hemodynamic stability.

To confirm previous case reports on perioperative management in a neonatal patient undergoing adrenal tumor resection to treat Cushing syndrome, we performed a PubMed search using the following search terms using the PubMed: (1) (Cushing syndrome) AND (anesthesia OR anesthetic OR perioperative OR hemodynamic) AND (infant OR neonate OR child), and (2) (Cushing syndrome) AND (left ventricular outflow tract obstruction OR LVOTO) AND (infant OR neonate OR child). No case report on perioperative management was found in this search on June 25, 2026.

In conclusion, we present a case of general anesthesia in a 6-month-old boy with ACTH-independent Cushing syndrome and rarely occurring LVOTO. Preoperative fluid therapy and perioperative hydrocortisone administration may contribute to hemodynamic stability during surgery for left ventricular hypertrophy and LVOTO caused by hypercortisolism and hypertension. Sevoflurane quickly decreased severe reactive hypertension during manipulation of the adrenal tumor. A preoperative echocardiogram is essential to check the presence of LVOTO, which requires preoperative volume management.

## Supplementary Information

Supplementary Material 1.

## Data Availability

Data sharing does not apply to this article as no datasets were generated or analyzed during the current study.

## Abbreviations

ACTH: Adrenocorticotropic hormone
LVOTO: Left ventricular outflow tract obstruction
TTE: Transthoracic echocardiography
SBP: Systolic blood pressure
PEEP: Positive end-expiratory pressure
POD: Postoperative day
ICU: Intensive care unit
TEE: Transesophageal echocardiography
