# Supplementary material for: Hemodynamic management, including for left ventricular outflow tract obstruction, in an infant with ACTH-independent Cushing syndrome undergoing adrenal tumor removal: a case report
Source: JA Clin Rep. 2026 Jul 11;12:33. doi: 10.1186/s40981-026-00868-2 (PMC13638925; doi:10.1186/s40981-026-00868-2)
Supplement: Supplementary file 1 [file 40981_2026_868_MOESM1_ESM.docx]

**CARE Checklist of information to include when writing a case report**

| **Topic** | **Item No** | **Checklist item description** | **Reported on Page Number/Line Number** |
| --- | --- | --- | --- |
| Title | 1 | The diagnosis or intervention of primary focus followed by the words “case report” | P.1, L.1-3 |
| Key Words | 2 | 2 to 5 key words that identify diagnoses or interventions in this case report, including "case report" | P.1, L.13-14 |
| Abstract  (Structured summary) | 3a | Background: state what is known and unknown; why the case report is unique and what it adds to existing literature. | P.2, L2-5 |
|  | 3b | Case Description: describe the patient’s demographic details, main symptoms, history, important clinical findings, the main diagnosis, interventions, outcomes and follow-ups. | P.2, L.6-12 |
|  | 3c | Conclusions: summarize the main take-away lesson, clinical impact and potential implications. | P.2, L.13-15 |
| Introduction | 4 | One or two paragraphs summarizing why this case is unique **(may include references)** | P.3, L.2-24 |
| Patient Information | 5a | De-identified patient specific information | P.4, L2-3; figure 1 |
|  | 5b | Primary concerns and symptoms of the patient | P.4, L.3-7 |
|  | 5c | Medical, family, and psycho-social history including relevant genetic information | N/A |
|  | 5d | Relevant past interventions with outcomes | P.4, L.8-10 |
| Clinical Findings | 6 | Describe significant physical examination (PE) and important clinical findings | P.4, L.2-8 |
| Timeline | 7 | Historical and current information from this episode of care organized as a timeline | N/A |
| Diagnostic Assessment | 8a | Diagnostic testing (such as PE, laboratory testing, imaging, surveys). | P.4, L5-7 |
|  | 8b | Diagnostic challenges (such as access to testing, financial, or cultural) | N/A |
|  | 8c | Diagnosis (including other diagnoses considered) | N/A |
|  | 8d | Prognosis (such as staging in oncology) where applicable | N/A |
| Therapeutic Intervention | 9a | Types of therapeutic intervention (such as pharmacologic, surgical, preventive, self-care) | P.4, L9-10 |
|  | 9b | Administration of therapeutic intervention (such as dosage, strength, duration) | P.4, L.12-P.5, L.17 |
|  | 9c | Changes in therapeutic intervention (with rationale) | P.5, L17-20 |

| Follow-up and Outcomes | 10a | Clinician and patient-assessed outcomes (if available) | P.5, L.21-25 |
| --- | --- | --- | --- |
|  | 10b | Important follow-up diagnostic and other test results | P.6, L1-L.7 |
|  | 10c | Intervention adherence and tolerability (How was this assessed?) | N/A |
|  | 10d | Adverse and unanticipated events | P.5, L.17-19 |
| Discussion | 11a | A scientific discussion of the strengths AND limitations associated with this case report | P.7, L.2-P.8, L.11 |
|  | 11b | Discussion of the relevant medical literature **with references** | P.7, L.23-25  P.8, L1-2 |
|  | 11c | The scientific rationale for any conclusions (including assessment of possible causes) | P.7, L13-17 |
|  | 11d | The primary “take-away” lessons of this case report (without references) in a one paragraph conclusion | P.8, L.13-19 |
| Patient Perspective | 12 | The patient should share their perspective in one to two paragraphs on the treatment(s) they received | N/A |
| Informed Consent | 13 | Did the patient give informed consent? Please provide if requested | **Yes** |

Please leave this space alone as it will be supplemented by the editorial office when needed.
